# Machine learning applied to near-infrared spectra for clinical pleural effusion classification

**DOI:** 10.1038/s41598-021-87736-4

**Published:** 2021-05-03

**Authors:** Zhongjian Chen, Keke Chen, Yan Lou, Jing Zhu, Weimin Mao, Zhengbo Song

**Affiliations:** 1 Cancer Hospital of the University of Chinese Academy of Sciences, Chinese Academy of Sciences, Banshandong Road#1, Hangzhou, 310000 Zhejiang Province China; 2 Zhejiang Cancer Hospital, Banshandong Road#1, Hangzhou, 310000 Zhejiang Province China; 3 Institute of Cancer and Basic Medicine (IBMC), Chinese Academy of Sciences, Hangzhou, China; 4 College of Pharmaceutical Sciences, Zhejiang University, Yuhangtang Road#866, Hangzhou, 310000 Zhejiang Province China; 5 Intensive Care Unit, Zhejiang Medical & Health Group Hangzhou Hospital, Banshan Kangjian Road #1, Hangzhou, 310000 Zhejiang Province China

**Keywords:** Classification and taxonomy, Machine learning

## Abstract

Lung cancer patients with malignant pleural effusions (MPE) have a particular poor prognosis. It is crucial to distinguish MPE from benign pleural effusion (BPE). The present study aims to develop a rapid, convenient and economical diagnostic method based on FTIR near-infrared spectroscopy (NIRS) combined with machine learning strategy for clinical pleural effusion classification. NIRS spectra were recorded for 47 MPE samples and 35 BPE samples. The sample data were randomly divided into train set (n = 62) and test set (n = 20). Partial least squares, random forest, support vector machine (SVM), and gradient boosting machine models were trained, and subsequent predictive performance were predicted on the test set. Besides the whole spectra used in modeling, selected features using SVM recursive feature elimination algorithm were also investigated in modeling. Among those models, NIRS combined with SVM showed the best predictive performance (accuracy: 1.0, kappa: 1.0, and AUC_ROC_: 1.0). SVM with the top 50 feature wavenumbers also displayed a high predictive performance (accuracy: 0.95, kappa: 0.89, AUC_ROC_: 0.99). Our study revealed that the combination of NIRS and machine learning is an innovative, rapid, and convenient method for clinical pleural effusion classification, and worth further evaluation.

## Introduction

Pleural effusion is an abnormal accumulation of fluid in the pleural cavity. Two types of pleural effusions in clinics are: (1) transudative pleural effusion, resulting from fluid leaking into the pleural cavity, commonly caused by heart failure, cirrhosis, and post-surgery; (2) exudative pleural effusion, resulting from leaky blood vessels, mainly caused by cancer^1,2^, tuberculosis^3^, pulmonary embolism^4^, and pneumonia^5^. Exudative pleural effusion can be further classified as malignant or benign based on the detection of malignant cells in the pleural fluid. Pleural fluid of malignant pleural effusions (MPE) contains cancer cells, while benign pleural effusions (BPE) does not^6^. An accurate diagnosis of MPE is crucial, since MPE can be an indication of pleural metastases caused by lung cancer, breast cancer, ovarian cancer and lymphomas, with lung cancer being the leading cause^7–9^. Lung cancer patients with complicated MPE are usually classified as stage IV, and facing significantly different treatments and poor prognosis from those without MPE^10^. In the meantime, patients of pulmonary tuberculosis also show similar exudative effusions and overlapping symptoms as lung cancer, including short breath, chronic cough, fatigue and unexplained weight loss, making accurate diagnosis of MPEs more important and challenging^8^.

Current standard MPE diagnosis methods, including cytological and histological examinations, are not applicable for all cases. On the one hand, samples are hardly collected from malignant cells or tissues^1^. On the other hand, diagnoses for pulmonary tuberculosis usually need bacterial culture, which is time-consuming. Therefore, the undiagnosed effusion—especially when it is an undiagnosed MPE—may delay the treatment of lung cancer. Many cancer biomarkers, such as CEA, CA125, CA15-3, CA19-9, CYFRA21-1, and VEGF were investigated to help diagnose MPE. However, the sensitivity of the existing biomarkers was low: 54% for CEA, 48% for CA125, 51% for CA15-3, 25% for CA19-9, 55% for CYFRA21-1, 75% for VEGF^9,11–15^. Therefore, an innovative diagnostic technique with a better sensitivity is needed.

Near-infrared spectroscopy (NIRS) is a spectroscopic tool using the near-infrared region of the electromagnetic spectrum from 780 to 2500 nm (4000 cm^−1^ to 12,820 cm^−1^)^16^. NIRS has been utilized as a fast, non-invasive tool for disease diagnosis, including cancer diagnosis, due to its ability of reflecting changes in molecular compositions by identifying different bonds vibrations in functional groups^17–20^. The variations in metabolites between MPE and BPE have been revealed by past metabonomic studies, indicated an increased amount of valine, lactate, alanine, lipids, and free fatty acids (FFAs) (16:0, 18:0, and 18:1) along with a decreased amount of acetoacetate, creatinine, β-glucose, and α-glucose in MPE^1,8,21^. In addition, our previous metabolomics results revealed that the metabolites composition, such as lipids and oxidized polyunsaturated fatty acids, varies between MPE and BPE. Therefore, NIRS might be able to distinguish the differences between the chemical compositions of MPE and BPE, and contribute to a novel diagnosis method with a higher sensitivity.

In the present study, a total of 82 pleural effusion samples were analyzed, including 47 MPE samples from diagnosed lung adenocarcinoma patients and 35 BPE samples from patients with diagnosed tuberculosis or tuberculous pleurisy. NIRS technology combined with machine learning approaches, including partial least squares (PLS), random forest (RF), support vector machine (SVM), and gradient boosting machine (GBM) models, were used to screen for the characteristics in near-infrared spectra between MPE and BPE samples.

## Materials and methods

### Pleural effusion samples

A total of 82 pleural effusion samples were obtained from the biobank of Zhejiang Cancer Hospital in Hangzhou, China. MPE samples were collected from 47 patients diagnosed with lung adenocarcinoma, complicated with pleural metastases. BPE samples were collected from 35 patients diagnosed with pulmonary tuberculosis and/or tuberculous pleurisy. Informed consent was obtained from all individual participants included in the study, and our study was approved by the Ethics Committee of Zhejiang Cancer Hospital. All methods were performed in accordance with the relevant guidelines and regulations. The diagnoses were based on cytological or histological examinations for MPE and bacterial culture which were performed in the cases of tuberculosis. All the pleural effusion samples were spun at 1600 g for 10 min at 4 °C, and the aliquot of supernatant was stored at -80 °C until analysis. Basic information of patients, including gender, age, and pathological information, were collected (Table 1).

**Table 1:** Demographic and clinical characteristics of malignant pleural effusion (MPE) and benign pleural effusion (BPE) cases.

Characteristics	MPE (n = 47)	BPE (n = 35)
Age, years		
	64 ± 10	49 ± 19
Gender		
Male	27 (57.4%)	26 (74.3%)
Female	20 (42.6%)	9 (25.7%)
Cause	Lung adenocarcinoma	Tuberculosis/Tuberculous pleurisy

### NIRS analysis and spectra collection

The frozen samples were thawed to room temperature before analysis. The NIR spectra of the pleural effusions were collected using an Antaris™ II FT-NIR analyzer (Thermo Nicolet, USA) with air as a reference. Aquartz colorimetric tube with an optical path of 2 mm was used as the sample cup. Each spectrum was obtained from 32 successive scans from 4000 to 10,000 cm^−1^ with a spectral resolution of 4 cm^−1^. The spectrum was recorded by absorbance. Each sample was analyzed in triplicate and the average spectrum was calculated by TQ Analyst 8.0 data processing software.

### Data analysis

#### Randomly slicing the data into train set and test set and preprocess

This cohort was randomly split into a train set of 62 cases (36 MPE and 26 BPE) and test set of 20 cases (11 MPE and 9 BPE) using *“sample”* function in R.

#### Preprocess

Spectra data in train set were mean-centered, scaled to unit variance, smoothed using a Savitzky-Golay filter, and dimensionally reduced by PCA analysis via generation of audit data summarizing discrete variables. Spectra in test set were preprocessed with the same method and the same values of parameters in train set.

#### Model training and testing

For PLS, RF, and GBM, model training and parameter tuning were conducted with *caret* R package, in which 10 repeated, fivefold cross validation was used. For SVM, model training was performed using *e1071* R package with fivefold cross validation. Accuracy was used to select the optimal model by the largest value. The running time for each model was measured in the following CPU condition: Intel(R) Core (TM) i5-8250U CPU@ 1.60 GHz.

#### Feature wavelength selection with SVM-RFE algorithm

SVM-RFE algorithm was used to rank the wavenumbers in train set. The algorithm processes were briefly described as follows: (1) train the SVM model; (2) compute the weight vector; (3) rank the variables from the minimum to the maximum by square weights; (4) update the feature ranking list; (5) eliminate the feature with the smallest square weight, and repeat from Step 1 until all the features were ranked. In order to optimize the subset size of the features, a series of subsets with different sizes of wavenumber (from top 1 to the total number) were evaluated for their predictive performances.

## Results

### NIR spectral analysis

**Figure 1:**
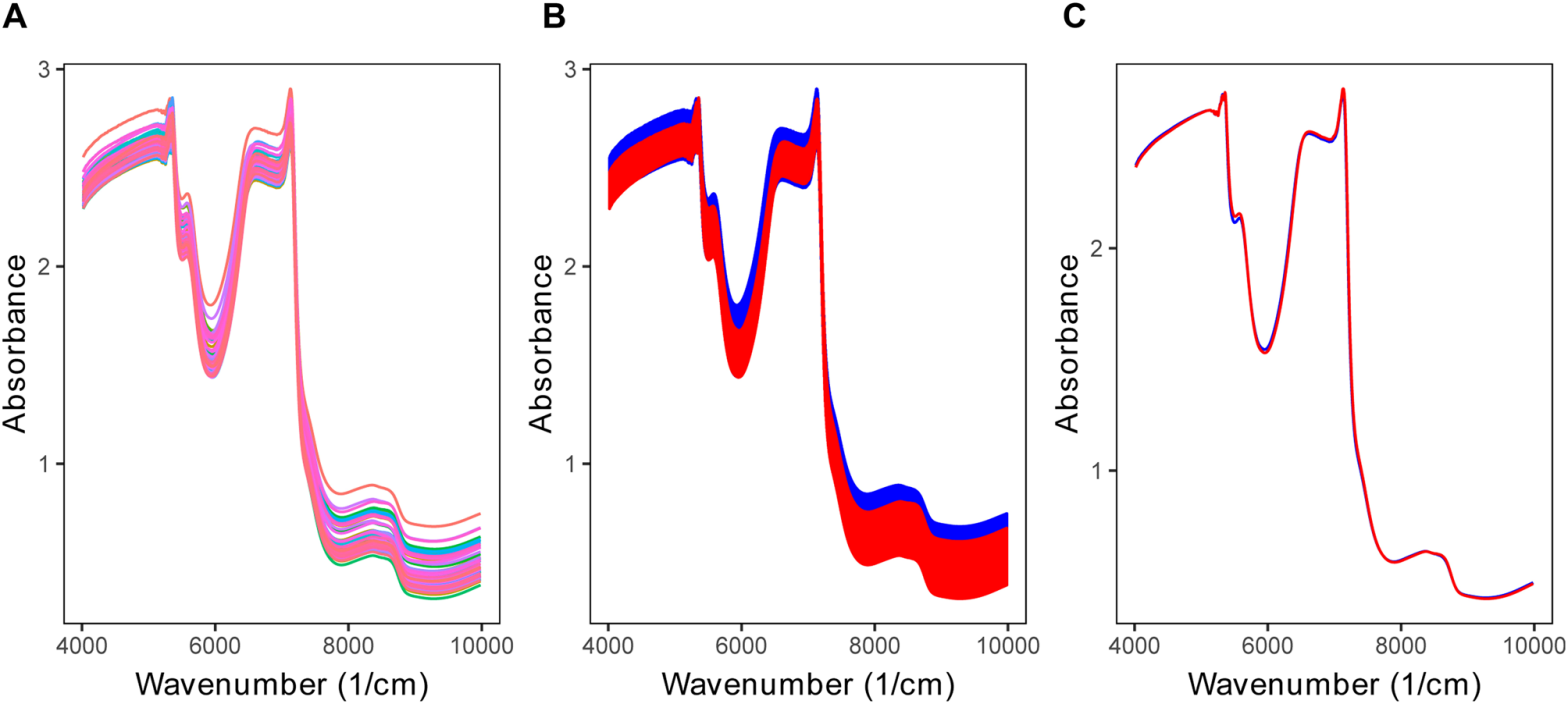
Raw NIR spectra of 82 pleural effusion samples (A), color-label MPE (red) and BPE (blue) NIR spectra (**B**), and the average NIR spectra for MPE (red) and BPE (blue) samples.

Plots of the raw NIR spectra of the 82 pleural effusions, groups of MPE and BPE samples, and their average spectra were illustrated in Fig. 1. Evidently, due to the broad and overlapping spectra peak, there was no significant difference between MPE and BPE samples in raw spectra, and the direct interpretation is nearly impossible. However, though there were no feature peaks, the NIR spectra still contain a lot of information in terms of the chemical composition of pleural effusion. There are four regions referring to different chemical substructures: wavenumbers between 4200 and 5500 cm^−1^ indicate the CH, OH and NH stretch/CH deformations in the phenyl; between 5400 and 6100 cm^−1^ refers to the first overtone of CH; wavenumbers of 6200 to 7600 cm^−1^ indicate the first overtone of OH, NH, and CH; and wavelengths of 7900 to 9000 cm^−1^ indicate the second overtone of CH. NH, and CH combinations were denoted by wavenumbers of 6200 to 7600 cm^−1^; and the second overtone of CH was denoted by wavelengths of 7900 to 9000 cm^−1^^17,22^.

### Principal component analysis

**Figure 2:**
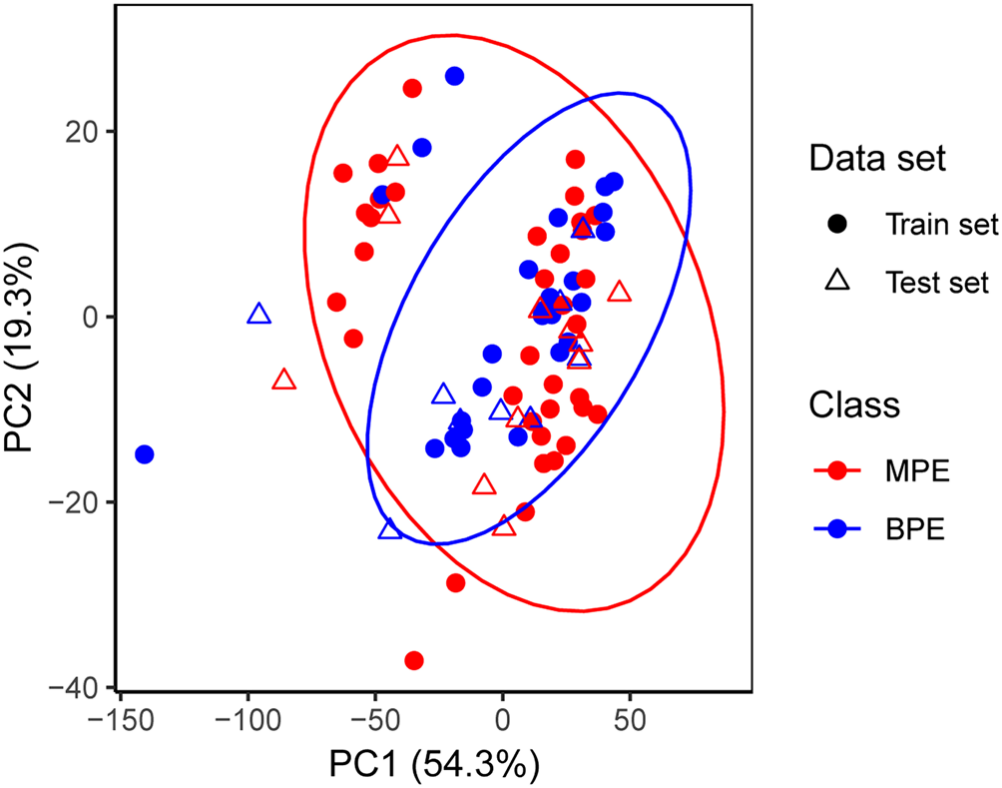
Scatter plots of the first two principal components (PCs). The variance explained PC1 and PC2 were 54.3% and 19.3%, respectively. 82 cases were randomly split into train set (n = 62, filled circle) and test set (n = 20, hollow triangle). 47 MPE cases are colored red, and 35 BPE cases are colored blue.

As an unsupervised model, a principal component analysis (PCA) was performed to check the extent of clustering of the samples and to investigate the potential NIR features for differentiating between MPE and BPE classes. Figure 2 shows a scatter plot of the first two principal components (PCs), accounting for about 94.7% of the total variation. However, there was no clear separation between MPE and BPE samples, which indicated that the structure or the relationship of the data might be complicated, non-linear, and therefore unfit for an unsupervised model.

### Predictive performances in pleural effusion classification

**Figure 3:**
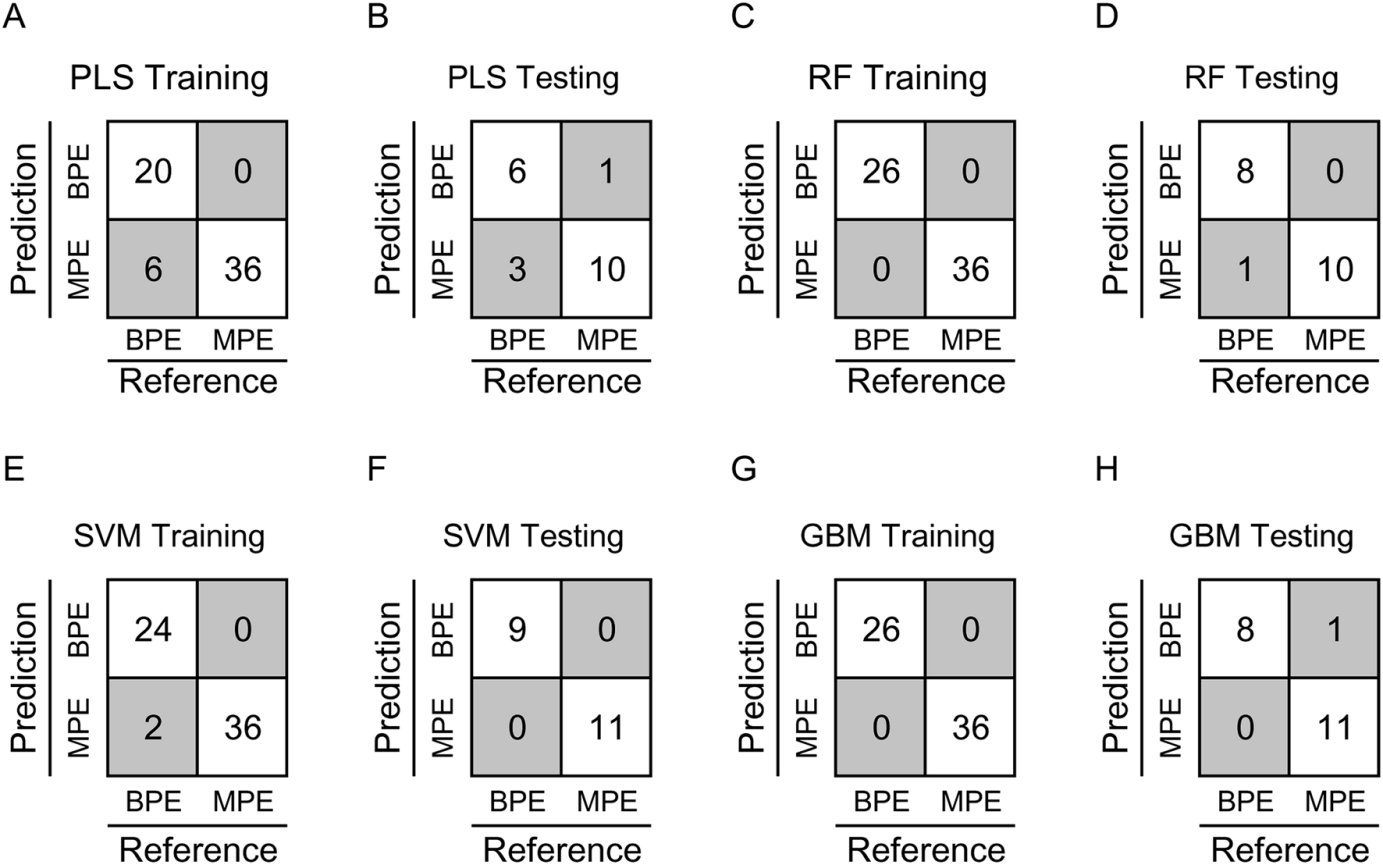
Predictive performance of the 4 models in train set and test set. PLS modeling in train set (**A**) and test set (**B**); RF modeling in train set (**C**) and test set (**D**); SVM modeling in train set (**E**) and test set (**F**); GBM modeling in train set (**G**) and test set (**H**). Good prediction is defined when predictive class is the same as the reference (no background), otherwise bad prediction is defined (gray background).

**Figure 4:**
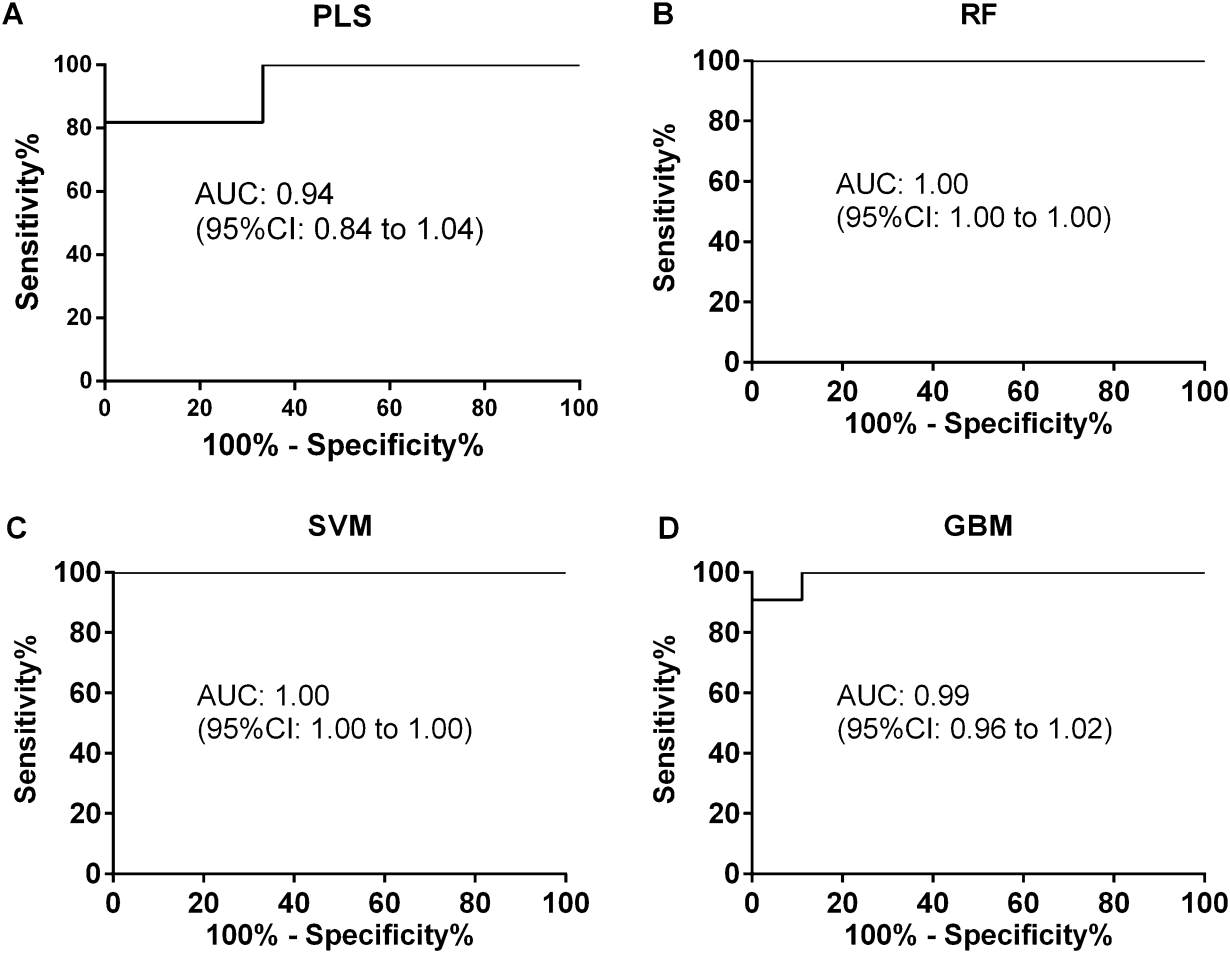
Receiver operating curve (ROC) in predicting pleural effusion classes for the test set. (**A**) Result from PLS model; (**B**) result from RF model; (**C**) result from SVM model; (**D**) result from GBM model.

For PLS model, the optimized number of components used in model was 3, and the time for running the model is 1.05 s. The predictive accuracy, kappa, and AUC_ROC_ in the test set were 0.91, 0.67, and 0.94, respectively (Figs. 3A,B, 4A; Table 2).

**Table 2:** Predictive performance of the 4 machine learning approaches in training and testing sets in the present study.

Data set	Performance^a^	PLS^b^	RF^c^	SVM^d^	GBM^e^
Train set (n = 62)	Accuracy	0.90	1.00	0.96	1.00
	Kappa	0.79	1.00	0.93	1.00
	Sensitivity	1.00	1.00	1.00	1.00
	Specificity	0.77	1.00	0.92	1.00
Test set (n = 20)	Accuracy	0.80	0.95	1.00	0.95
	Kappa	0.59	0.90	1.00	0.90
	Sensitivity	0.91	1.00	1.00	0.91
	Specificity	0.67	0.89	1.00	1.00

^a^Predictive performance was calculated using confusionMatrix function from *caret* package in R.

^b^Partial least squares, PLS.

^c^Random forest, RF.

^d^Support vector machines, SVM.

^e^Gradient boosting machine, GBM; PLS, RF, and GBM modeling were performed using *caret* R package, and SVM modeling was conducted using *e1071* R package.

For RF model, the optimized value for mtry is 32, and the time used for running the model is 8.25 s. The predictive accuracy, kappa, and AUC_ROC_ in the test set were 0.95 and 0.90, 1.00, respectively (Figs. 3C,D, 4B; Table 2).

SVM model performs best with “linear” kernel, cost value of 5 and number of support vectors of 19, and the running time is 0.52 s. The predictive accuracy, kappa, and AUC_ROC_ in test were 1.00, 1.00, and 1.00, respectively (Figs. 3E,F, 4C; Table 2).

For GBM model, the final optimized model was with the following parameters: ntrees value of 50, interaction depth value of 1, shrinkage value of 0.1 and n minobsinnode value of 10. The running time was 5.57 s. The predictive accuracy, kappa, and AUC_ROC_ were 0.95, 0.9, and 0.99, respectively (Figs. 3G,H, 4D; Table 2).

Among the four models, the performance of PLS was unsatisfactory. RF and GBM have exhibited relatively high accuracy and kappa values in both train and test sets, but with a relatively longer computational time. In contrast, SVM was the fastest model in computation and has displayed the best predictive performance in the test group. Therefore, SVM was considered as the best model for pleural effusion classification in this study. More detailed model performance parameters were illustrated in Table 2.

### Wavenumber selection

**Figure 5:**
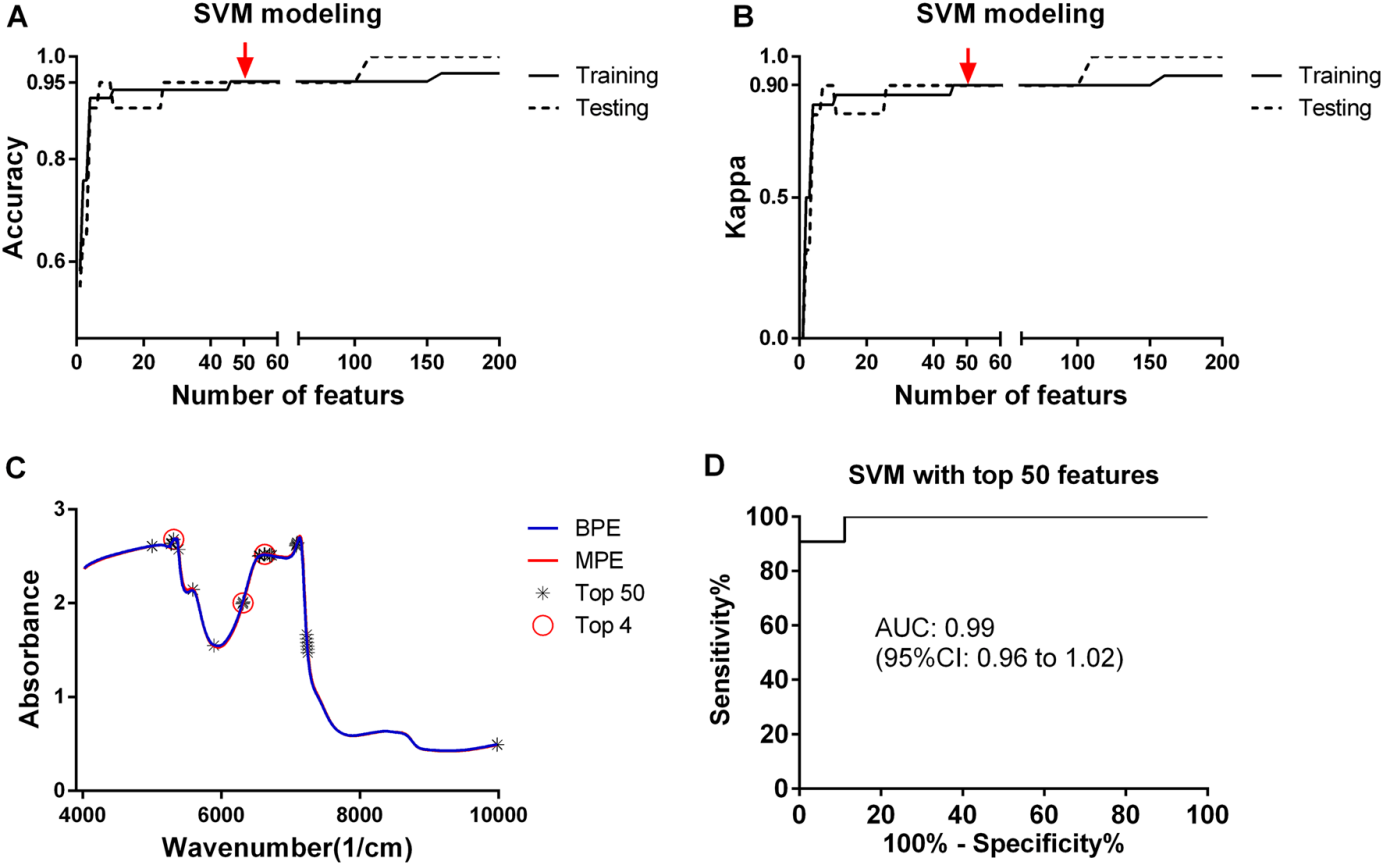
SVM modeling with different sizes of feature wavenumbers. The curves of predictive accuracy (**A**) and kappa (**B**) values increase as the sizes of top features grow. Top 50 feature wavenumbers are selected in final SVM, and the top 50 feature wavenumbers (including top 4) were marked on the average spectra; (D) ROC in predicting pleural effusion classes for the test set by SVM mode with top 50 features.

After ranking wavenumbers by SVM-RFE algorithm, SVM model with different sizes of featured wavenumbers was tested (from the top 1 to all the features). The results showed that the predictive accuracy increased dramatically, exceeding 0.90 (0.79 kappa) in both train and test sets within the size of 4, and then slowly reached 0.95 (0.89 kappa) at the size of 46 (Fig. 5A,B). The highest predictive accuracy reached 0.97 (0.93 kappa) (at the size of 160) in the train set, and 1.00 (1.00 kappa) (at the size of 102) in the test set. Finally, the top 50 featured wavenumbers were selected as variables in SVM model. Figure 5C displayed the distribution of the top 4 (6626 cm^−1^, 5311 cm^−1^, 6622 cm^−1^, and 6309 cm^−1^) and the top 50 featured wavenumbers. SVM model with the top 50 features had an AUC_ROC_ of 0.99 for predicting the pleural effusion classes (Fig. 5D).

## Discussion and conclusion

Our study applied several machining learning approaches in NIRS analysis to classify malignant and benign pleural effusion samples, through which a rapid, convenient and accurate diagnostic method was successfully developed.

The diagnostic performance of NIRS has been investigated in the past studies. For example, Chen et al. established a NIRS based method to distinguish between normal and malignant colorectal tissues^17^. However, to the best of our knowledge, our study is the first one that applyed NIRS to the classification of pleural effusion. MPE usually indicates advanced development in cancer, which contributes to a unique cancerous microenvironment that is significantly different from the surrounding healthy tissues, featured with variations in metabolites including proteins and lipids^1,8,10,21^. Therefore, NIRS can be used to distinguish the variation of chemicals in samples ^17–20^.

Although the NIRS of malignant and benign samples overlapped to a great extent, additional application of machine learning aided in the separation of malignant and benign samples and some spectral regions that are of high diagnostic values were detected. According to our previous metabolomics results using the same samples, malignant pleural effusion differs from benign samples in metabolites like acylcarnitines, oxidized polyunsaturated fatty acids (PUFAs), and ether lipids^23^. In line, the top 50 diagnostic wavenumbers detected by SVM-RFE denoted functional groups including CH, CH2, and CH3, NH, free and bound OH. The spectral intervals of CH2 and CH3 arisen from stretching vibrations at 5577 to 5889 cm^−1^ of the first overtone, and that of OH of stretching vibrations at 7077 to 7093 cm^−1^ and at 9977 to 9981 cm^−1^ denoted the change in ether lipids. In addition, CH group of combined vibrations of second overtone was detected at 7227 to 7247 cm^−1^ together with the aforementioned OH groups explained the existence of oxidized PUFAs. Acylcarnitines can also be annotated in terms of the NH (at 6306 to 6618 cm^−1^), CH, and OH detected.

Compared to the traditional diagnostic methods, such as cytological or histological examinations, our method is simpler and more convenient since the supernatant of the pleural effusion sample is the only need. Hence our method could be a supplementary tool when there are difficulties in collecting malignant cells or tissues. In addition, compared to the high throughput method, such as metabolomics, our NIRS method is more economical, less time- and labor-consuming, and needs no additional sample preparation. Altogether, our NIRS method is worthy to be further developed for clinical application.

At present, NIRS-SVM has been considered as the best model for pleural effusion classification. SVM algorithm is fast and has a high predictive performance for pleural effusion classification. Compared to models using the whole spectra, SVM with the top 50 features is less complex and more stable in application, and worthy to be further investigated.

The major limitation in our study is that our cohort size was relatively small. In addition, the types of pleural effusions were too limited. A larger cohort with more types of pleural effusions could be studied in the future. In conclusion, our study provided an idea that NIRS could be a helpful tool in the classification of pleural effusion, with advantages of high speed and accuracy, which might improve the current clinic diagnostic methods for MPE.

## Supplementary Information

Supplementary Information 1.

Supplementary Information 2.

Supplementary Information 3.
