# Supplementary material for: Machine learning applied to near-infrared spectra for clinical pleural effusion classification
Source: Sci Rep. 2021 May 3;11:9411. doi: 10.1038/s41598-021-87736-4 (PMC8093263; doi:10.1038/s41598-021-87736-4)
Supplement: Supplementary file 3 — Supplementary Information 3. [file 41598_2021_87736_MOESM3_ESM.docx]

**R codes for machine learning**

#R package loaded

library(e1071)

library(caret)

library(Rmisc)

#Randomly split 82 samples into train set (62 cases) and test set (20 cases)

#file “Near-infrared spectra data for R.csv”

dt0 <- read.csv(“path/Near-infrared spectra data for R.csv”)

train <- sample(82, size = 62)

train.set <- dt0[train,]

test.set <- dt0[-train,]

#Preprocess

preprocess <- preProcess(train.set[,-c(1,2,3)], method = c("center","scale"))

#scale with the same method and parameters

train.set[,-c(1,2,3)] <- predict(preprocess, train.set[,-c(1,2,3)])

test.set[,-c(1,2,3)] <- predict(preprocess, test.set[,-c(1,2,3)])

# PCA

PCA_train.set <- prcomp(train.set[,-c(1,2,3)], center = F, scale. = F)

PCA_test.set <- predict(PCA_train,test.set[,-c(1,2,3)])

#Machine learning

#For PLS, RF, and GBM, model training and parameter tuning were conducted with caret R #package, in which 10 repeated, 5-fold cross validation was used. For SVM model training #was performed using e1071 R package in which 5-fold cross validation was set.

# PLS, RF, and GBM modeling with *caret* R package

fitControl <- trainControl(## 5-fold CV, 10 repeats

method = "repeatedcv",

number = 5,

repeats = 10)

fit <- train(class ~ ., data = train.set[,-c(1,2,3)],

method = "gbm", # gbm, pls and rf

trControl = fitControl,

verbose = FALSE

)

confusionMatrix(predict(fit,test.set), as.factor(test.set$class))

#svm modeling with *e1071* R package

fit.svm <- svm(class ~ .,data = train.set[,-c(1,2,3)],

cross = 5,

kernel = "linear",

type = "C-classification",

scale =F,

probability= T,

cost = 5

)

confusionMatrix(predict(fit.svm, test.set), test.set$class)

#Feature wavelength selection with SVM-RFE algorithm

svmrfeFeatureRanking = function(x,y){

n = ncol(x)

survivingFeaturesIndexes = seq(1:n)

featureRankedList = vector(length=n)

rankedFeatureIndex = n

while(length(survivingFeaturesIndexes)>0){

#train the support vector machine

svmModel <- svm(x[, survivingFeaturesIndexes],

y,

cost = 5,

cachesize=500,

scale=FALSE,

type="C-classification",

kernel="linear" )

#compute the weight vector, and "%*%" standing for matrix multiplication.

w <- t(svmModel$coefs) %*% svmModel$SV #// svmModel$SV means SV value.

# Here, we also compute the b value in formula D(x)=sum(w*x)+b.

# b <- -svmModel$rho

#compute ranking criteria

rankingCriteria <- w * w

#rank the features from minimum to maximum.

ranking <- sort(rankingCriteria, index.return = TRUE)$ix

#update feature ranked list

#// and take the smallest-ranked one into the ranklist backwards.

featureRankedList[rankedFeatureIndex] <- survivingFeaturesIndexes[ranking[1]]

rankedFeatureIndex <- rankedFeatureIndex - 1

#eliminate the feature with smallest ranking criterion

(survivingFeaturesIndexes <- survivingFeaturesIndexes[-ranking[1]])

}

return (featureRankedList)

}

SVM_RFE_res <- svmrfeFeatureRanking(data,as.numeric(labels))

data_svmrfe <- data[SVM_RFE_res,]

write.csv(data_svmrfe, file="data_svmrfe.csv")
